# Thio­phene-2-carbaldehyde 2,4-dinitro­phenyl­hydrazone

**DOI:** 10.1107/S1600536808038397

**Published:** 2008-11-22

**Authors:** Zhi-gang Yin, Heng-yu Qian, He-ping Li, Jie Hu, Chun-xia Zhang

**Affiliations:** a Key Laboratory of Surface and Interface Science of Henan, School of Materials and Chemical Engineering, Zhengzhou University of Light Industry, Zhengzhou 450002, People’s Republic of China; b Department of Materials and Chemical Engineering, Guiling University of Technology, People’s Republic of China

## Abstract

In the approximately planar molecule of the title compound, C_11_H_8_N_4_O_4_S, the dihedral angle between the thio­phene and benzene rings is 5.73 (10)°. In the crystal structure, bifurcated inter/intra­molecular N—H⋯(O,O) hydrogen bonds are present. The intermolecular links lead to inversion dimers containing an *R*_2_^2^(12) graph-set motif.

## Related literature

For general background, see: Okabe *et al.* (1993 ▶). For graph-set notation, see: Etter *et al.* (1990 ▶); Bernstein *et al.* (1995 ▶).
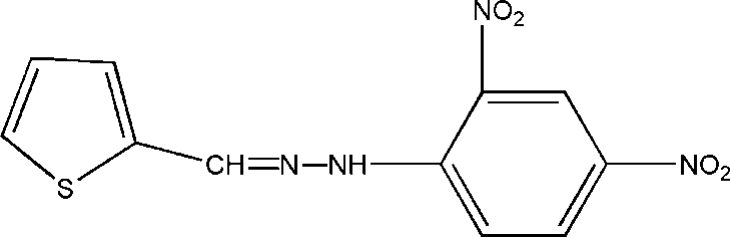


## Experimental

### 

#### Crystal data

C_11_H_8_N_4_O_4_S
*M* *_r_* = 292.27
Monoclinic, 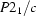

*a* = 4.8994 (17) Å
*b* = 9.520 (3) Å
*c* = 25.708 (8) Å
β = 92.71 (2)°
*V* = 1197.7 (7) Å^3^
*Z* = 4
Mo *K*α radiation
μ = 0.29 mm^−1^
*T* = 291 (2) K
0.30 × 0.26 × 0.24 mm

#### Data collection

Bruker SMART APEX CCD diffractometer
Absorption correction: multi-scan (*SADABS*; Bruker, 2000 ▶) *T*_min_ = 0.915, *T*_max_ = 0.929
11010 measured reflections
2285 independent reflections
1718 reflections with *I* > 2σ(*I*)
*R*_int_ = 0.047

#### Refinement

*R*[*F* ^2^ > 2σ(*F* ^2^)] = 0.045
*wR*(*F* ^2^) = 0.087
*S* = 1.02
2285 reflections
184 parameters
H atoms treated by a mixture of independent and constrained refinement
Δρ_max_ = 0.16 e Å^−3^
Δρ_min_ = −0.19 e Å^−3^

### 

Data collection: *SMART* (Bruker, 2000 ▶); cell refinement: *SAINT* (Bruker, 2000 ▶); data reduction: *SAINT*; program(s) used to solve structure: *SHELXTL* (Sheldrick, 2008 ▶); program(s) used to refine structure: *SHELXTL*; molecular graphics: *SHELXTL* and *PLATON* (Spek, 2003 ▶); software used to prepare material for publication: *SHELXTL*.

## Supplementary Material

Crystal structure: contains datablocks global, I. DOI: 10.1107/S1600536808038397/dn2406sup1.cif

Structure factors: contains datablocks I. DOI: 10.1107/S1600536808038397/dn2406Isup2.hkl

Additional supplementary materials:  crystallographic information; 3D view; checkCIF report

## Figures and Tables

**Table 1:** Hydrogen-bond geometry (Å, °)

*D*—H⋯*A*	*D*—H	H⋯*A*	*D*⋯*A*	*D*—H⋯*A*
N3—H1⋯O1^i^	0.87 (2)	2.57 (2)	3.338 (2)	148.2 (19)
N3—H1⋯O1	0.87 (2)	2.01 (2)	2.630 (2)	127.4 (19)

Symmetry code: (i) 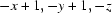.

**Fig. 1.**
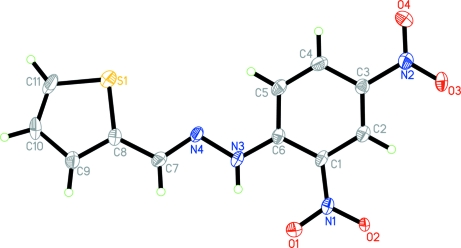
Molecular view of the title compound with the atom-labeling scheme. Displacement ellipsoids are drawn at the 30% probability level. H atoms are represented as small spheres of arbitrary radii.

**Fig. 2.**
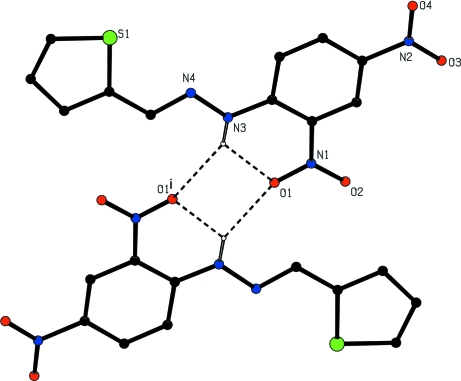
View showing the N-H···O hydrogen bonds buiding a R22(12) graph set motif. Hydrogen bonds are shown as dashed lines.

## supplementary crystallographic information

### Comment

2,4-Dinitrophenylhydrazine is a reagent which is widely used for condensation with aldehydes and ketones. Several phenylhydrazone derivatives have been shown to be potentially DNA-damaging and are mutagenic agents(Okabe *et al.* 1993). As part of our work, we have synthesized the title compound (I) and reported its cyrstal structure.

The title molecule is roughly planar. Indeed, the benzene and the thiophene rings are only slight twisted, making a dihedral angle of 5.73 (10)° (Fig. 1). The two nitro groups, O1/N1/O2 and O3/N2/O4 are coplanar with the benzene ring to which they are attached.

Intermolecular N-H···O hydrogen bonds link the molecule two by two around inversion center, building then a R_2_^2^(12) graph set motif (Etter *et al.*, 1990; Bernstein *et al.*, 1995) if the intramolecular N-H···O hydrogen bonds are ignored (Table 1, Fig.2).

### Experimental

2,4-dinitrophenylhydrazine(1 mmol, 0.198 g) was dissolved in anhydrous methanol, H_2_SO_4_ (98% 0.5 ml) was added to this, the mixture was stirred for several minitutes at 351 K, thiophene-2-carbaldehyde (1 mmol 0.112 g) in methanol (8 ml) was added dropwise and the mixture was stirred at refluxing temperature for 2 h. The product was isolated and recrystallized in methanol, purple single crystals of (I) was obtained after 5 d.

### Refinement

All H atoms were placed in calculated positions and treated as riding with C—H=0.93Å (aromatic) and N—H=0.86Å with *U*_iso_(H)=1.2*U*_eq_(C or N).

### Crystal data

**Table:**

C_11_H_8_N_4_O_4_S	*F*_000_ = 600
*M**_r_* = 292.27	*D*_x_ = 1.621 Mg m^−^^3^
Monoclinic, *P*2_1_/*c*	Mo *K*α radiation λ = 0.71073 Å
Hall symbol: -P 2ybc	Cell parameters from 5391 reflections
*a* = 4.8994 (17) Å	θ = 2.3–27.4º
*b* = 9.520 (3) Å	µ = 0.29 mm^−^^1^
*c* = 25.708 (8) Å	*T* = 291 (2) K
β = 92.71 (2)º	Block, purple
*V* = 1197.7 (7) Å^3^	0.30 × 0.26 × 0.24 mm
*Z* = 4	

### Data collection

**Table:**

Bruker SMART APEX CCD diffractometer	2285 independent reflections
Radiation source: sealed tube	1718 reflections with *I* > 2σ(*I*)
Monochromator: graphite	*R*_int_ = 0.047
*T* = 291(2) K	θ_max_ = 26.0º
φ and ω scans	θ_min_ = 1.6º
Absorption correction: multi-scan(SADABS; Bruker, 2000)	*h* = −6→5
*T*_min_ = 0.915, *T*_max_ = 0.929	*k* = −11→11
11010 measured reflections	*l* = −31→29

### Refinement

**Table:**

Refinement on *F*^2^	Secondary atom site location: difference Fourier map
Least-squares matrix: full	Hydrogen site location: inferred from neighbouring sites
*R*[*F*^2^ > 2σ(*F*^2^)] = 0.045	H atoms treated by a mixture of independent and constrained refinement
*wR*(*F*^2^) = 0.087	*w* = 1/[σ^2^(*F*_o_^2^) + (0.04*P*)^2^] where *P* = (*F*_o_^2^ + 2*F*_c_^2^)/3
*S* = 1.02	(Δ/σ)_max_ < 0.001
2285 reflections	Δρ_max_ = 0.16 e Å^−^^3^
184 parameters	Δρ_min_ = −0.19 e Å^−^^3^
Primary atom site location: structure-invariant direct methods	Extinction correction: none

### Special details

**Table:**

Geometry. All e.s.d.'s (except the e.s.d. in the dihedral angle between two l.s. planes) are estimated using the full covariance matrix. The cell e.s.d.'s are taken into account individually in the estimation of e.s.d.'s in distances, angles and torsion angles; correlations between e.s.d.'s in cell parameters are only used when they are defined by crystal symmetry. An approximate (isotropic) treatment of cell e.s.d.'s is used for estimating e.s.d.'s involving l.s. planes.
Refinement. Refinement of *F*^2^ against ALL reflections. The weighted *R*-factor *wR* and goodness of fit *S* are based on *F*^2^, conventional *R*-factors *R* are based on *F*, with *F* set to zero for negative *F*^2^. The threshold expression of *F*^2^ > σ(*F*^2^) is used only for calculating *R*-factors(gt) *etc*. and is not relevant to the choice of reflections for refinement. *R*-factors based on *F*^2^ are statistically about twice as large as those based on *F*, and *R*- factors based on ALL data will be even larger.

### Fractional atomic coordinates and isotropic or equivalent isotropic displacement parameters (Å^2^)

**Table:**

	*x*	*y*	*z*	*U*_iso_*/*U*_eq_	
C1	0.1154 (4)	0.2360 (2)	0.04553 (7)	0.0342 (4)	
C2	−0.0759 (4)	0.1287 (2)	0.04720 (7)	0.0350 (4)	
H2	−0.2010	0.1150	0.0193	0.042*	
C3	−0.0801 (4)	0.04351 (18)	0.08984 (7)	0.0315 (4)	
C4	0.1065 (4)	0.0673 (2)	0.13207 (7)	0.0383 (5)	
H4	0.1048	0.0087	0.1610	0.046*	
C5	0.2882 (4)	0.1738 (2)	0.13141 (7)	0.0350 (4)	
H5	0.4096	0.1870	0.1599	0.042*	
C6	0.2980 (4)	0.26512 (18)	0.08856 (7)	0.0291 (4)	
C7	0.8165 (4)	0.49823 (19)	0.13079 (7)	0.0389 (5)	
H7	0.7974	0.5614	0.1033	0.047*	
C8	1.0163 (4)	0.52284 (19)	0.17283 (7)	0.0341 (4)	
C9	1.1994 (5)	0.6353 (2)	0.17481 (8)	0.0418 (5)	
H9	1.2066	0.7050	0.1496	0.050*	
C10	1.3738 (5)	0.6289 (2)	0.22076 (9)	0.0465 (5)	
H10	1.5107	0.6939	0.2290	0.056*	
C11	1.3173 (5)	0.5174 (2)	0.25090 (8)	0.0522 (6)	
H11	1.4113	0.4975	0.2823	0.063*	
N1	0.0973 (4)	0.31477 (18)	0.00000 (6)	0.0394 (4)	
N2	−0.2778 (4)	−0.06932 (17)	0.09065 (7)	0.0414 (4)	
N3	0.4846 (4)	0.37052 (17)	0.08923 (6)	0.0378 (4)	
H1	0.483 (5)	0.426 (2)	0.0624 (9)	0.045*	
N4	0.6631 (3)	0.38663 (15)	0.13180 (6)	0.0321 (4)	
O1	0.2484 (4)	0.41150 (16)	−0.00353 (6)	0.0545 (4)	
O2	−0.0594 (3)	0.29004 (14)	−0.03604 (5)	0.0461 (4)	
O3	−0.4272 (3)	−0.08987 (16)	0.05310 (6)	0.0526 (4)	
O4	−0.2847 (3)	−0.13710 (15)	0.12986 (6)	0.0513 (4)	
S1	1.05706 (12)	0.41736 (6)	0.22559 (2)	0.04762 (18)	

### Atomic displacement parameters (Å^2^)

**Table:**

	*U*^11^	*U*^22^	*U*^33^	*U*^12^	*U*^13^	*U*^23^
C1	0.0302 (10)	0.0438 (10)	0.0277 (10)	−0.0016 (8)	−0.0096 (8)	−0.0034 (7)
C2	0.0330 (10)	0.0410 (10)	0.0303 (10)	−0.0007 (8)	−0.0046 (8)	−0.0039 (7)
C3	0.0326 (10)	0.0290 (8)	0.0325 (9)	−0.0001 (7)	−0.0024 (8)	−0.0025 (7)
C4	0.0414 (12)	0.0417 (10)	0.0314 (10)	0.0038 (8)	−0.0038 (9)	0.0075 (8)
C5	0.0259 (10)	0.0463 (10)	0.0323 (10)	0.0090 (8)	−0.0035 (8)	−0.0005 (8)
C6	0.0270 (9)	0.0339 (8)	0.0262 (9)	0.0002 (7)	−0.0004 (7)	−0.0062 (7)
C7	0.0510 (12)	0.0394 (10)	0.0257 (9)	−0.0026 (9)	−0.0057 (8)	−0.0013 (8)
C8	0.0316 (10)	0.0353 (9)	0.0349 (9)	−0.0023 (8)	−0.0025 (8)	−0.0079 (7)
C9	0.0473 (13)	0.0361 (9)	0.0412 (11)	−0.0042 (9)	−0.0073 (9)	−0.0059 (8)
C10	0.0387 (12)	0.0416 (10)	0.0581 (14)	−0.0045 (9)	−0.0105 (10)	−0.0220 (10)
C11	0.0629 (15)	0.0558 (13)	0.0357 (11)	0.0052 (11)	−0.0222 (11)	−0.0131 (9)
N1	0.0383 (10)	0.0452 (9)	0.0335 (9)	−0.0104 (8)	−0.0120 (8)	−0.0013 (7)
N2	0.0360 (10)	0.0421 (9)	0.0453 (10)	−0.0026 (7)	−0.0074 (8)	0.0047 (8)
N3	0.0387 (10)	0.0443 (9)	0.0290 (9)	−0.0077 (7)	−0.0126 (7)	−0.0001 (7)
N4	0.0345 (8)	0.0344 (8)	0.0266 (8)	−0.0003 (6)	−0.0087 (7)	−0.0040 (6)
O1	0.0659 (11)	0.0525 (9)	0.0435 (9)	−0.0225 (8)	−0.0163 (8)	0.0158 (7)
O2	0.0471 (9)	0.0505 (8)	0.0383 (8)	−0.0317 (7)	−0.0222 (7)	0.0182 (6)
O3	0.0488 (10)	0.0534 (9)	0.0537 (9)	−0.0260 (7)	−0.0185 (8)	−0.0002 (7)
O4	0.0566 (10)	0.0451 (8)	0.0519 (9)	−0.0169 (7)	−0.0008 (8)	0.0131 (7)
S1	0.0480 (3)	0.0553 (3)	0.0385 (3)	−0.0071 (2)	−0.0093 (2)	0.0040 (2)

### Geometric parameters (Å, °)

**Table:**

C1—C2	1.388 (3)	C8—C9	1.396 (3)
C1—N1	1.389 (2)	C8—S1	1.692 (2)
C1—C6	1.417 (2)	C9—C10	1.426 (3)
C2—C3	1.365 (3)	C9—H9	0.9300
C2—H2	0.9300	C10—C11	1.351 (3)
C3—C4	1.404 (2)	C10—H10	0.9300
C3—N2	1.447 (2)	C11—S1	1.696 (2)
C4—C5	1.350 (3)	C11—H11	0.9300
C4—H4	0.9300	N1—O1	1.188 (2)
C5—C6	1.406 (3)	N1—O2	1.1983 (19)
C5—H5	0.9300	N2—O4	1.199 (2)
C6—N3	1.357 (2)	N2—O3	1.199 (2)
C7—N4	1.302 (2)	N3—N4	1.377 (2)
C7—C8	1.442 (2)	N3—H1	0.87 (2)
C7—H7	0.9300		
			
C2—C1—N1	114.01 (15)	C9—C8—S1	112.01 (14)
C2—C1—C6	121.47 (17)	C7—C8—S1	123.66 (14)
N1—C1—C6	124.40 (17)	C8—C9—C10	110.86 (19)
C3—C2—C1	119.87 (17)	C8—C9—H9	124.6
C3—C2—H2	120.1	C10—C9—H9	124.6
C1—C2—H2	120.1	C11—C10—C9	112.19 (18)
C2—C3—C4	119.41 (17)	C11—C10—H10	123.9
C2—C3—N2	119.22 (15)	C9—C10—H10	123.9
C4—C3—N2	121.36 (16)	C10—C11—S1	113.08 (15)
C5—C4—C3	121.14 (18)	C10—C11—H11	123.5
C5—C4—H4	119.4	S1—C11—H11	123.5
C3—C4—H4	119.4	O1—N1—O2	118.16 (16)
C4—C5—C6	121.43 (17)	O1—N1—C1	117.89 (14)
C4—C5—H5	119.3	O2—N1—C1	123.94 (16)
C6—C5—H5	119.3	O4—N2—O3	123.26 (17)
N3—C6—C5	119.70 (15)	O4—N2—C3	117.21 (15)
N3—C6—C1	123.69 (17)	O3—N2—C3	119.53 (16)
C5—C6—C1	116.55 (17)	C6—N3—N4	119.65 (16)
N4—C7—C8	119.31 (16)	C6—N3—H1	117.6 (14)
N4—C7—H7	120.3	N4—N3—H1	122.8 (14)
C8—C7—H7	120.3	C7—N4—N3	114.87 (15)
C9—C8—C7	124.33 (18)	C8—S1—C11	91.85 (11)
			
N1—C1—C2—C3	180.00 (18)	C8—C9—C10—C11	−0.6 (3)
C6—C1—C2—C3	−3.8 (3)	C9—C10—C11—S1	0.1 (3)
C1—C2—C3—C4	1.3 (3)	C2—C1—N1—O1	177.51 (19)
C1—C2—C3—N2	−178.99 (18)	C6—C1—N1—O1	1.5 (3)
C2—C3—C4—C5	0.6 (3)	C2—C1—N1—O2	−3.3 (3)
N2—C3—C4—C5	−179.16 (18)	C6—C1—N1—O2	−179.4 (2)
C3—C4—C5—C6	0.1 (3)	C2—C3—N2—O4	−176.10 (19)
C4—C5—C6—N3	−179.66 (19)	C4—C3—N2—O4	3.6 (3)
C4—C5—C6—C1	−2.5 (3)	C2—C3—N2—O3	3.1 (3)
C2—C1—C6—N3	−178.60 (18)	C4—C3—N2—O3	−177.21 (19)
N1—C1—C6—N3	−2.8 (3)	C5—C6—N3—N4	0.2 (3)
C2—C1—C6—C5	4.3 (3)	C1—C6—N3—N4	−176.76 (17)
N1—C1—C6—C5	−179.88 (19)	C8—C7—N4—N3	−178.20 (17)
N4—C7—C8—C9	177.13 (19)	C6—N3—N4—C7	−173.88 (18)
N4—C7—C8—S1	−2.5 (3)	C9—C8—S1—C11	−0.66 (18)
C7—C8—C9—C10	−178.8 (2)	C7—C8—S1—C11	179.01 (19)
S1—C8—C9—C10	0.8 (2)	C10—C11—S1—C8	0.3 (2)

### Hydrogen-bond geometry (Å, °)

**Table:**

*D*—H···*A*	*D*—H	H···*A*	*D*···*A*	*D*—H···*A*
N3—H1···O1^i^	0.87 (2)	2.57 (2)	3.338 (2)	148.2 (19)
N3—H1···O1	0.87 (2)	2.01 (2)	2.630 (2)	127.4 (19)

Symmetry codes: (i) −*x*+1, −*y*+1, −*z*.

## References

[bb1] Bernstein, J., Davis, R. E., Shimoni, L. & Chang, N.-L. (1995). *Angew. Chem. Int. Ed. Engl.* **34**, 1555–1573.

[bb2] Bruker (2000). *SMART*, *SAINT* and *SADABS* Bruker AXS Inc., Madison, Wisconsin, USA.

[bb3] Etter, M. C., MacDonald, J. C. & Bernstein, J. (1990). *Acta Cryst.* B**46**, 256–262. 10.1107/s0108768189012929 2344397

[bb4] Okabe, N., Nakamura, T. & Fukuda, H. (1993). *Acta Cryst.* C**49**, 1678–1680.

[bb5] Sheldrick, G. M. (2008). *Acta Cryst.* A**64**, 112–122. 10.1107/S0108767307043930 18156677

[bb6] Spek, A. L. (2003). *J. Appl. Cryst.* **36**, 7–13.

